# A tendency to worse course of multisystem inflammatory syndrome in children with obesity: MultiOrgan Inflammatory Syndromes COVID-19 related study

**DOI:** 10.3389/fendo.2022.934373

**Published:** 2022-09-26

**Authors:** Aneta Monika Gawlik, Elżbieta Berdej-Szczot, Iga Chmiel, Miłosz Lorek, Aleksandra Antosz, Małgorzata Firek-Pędras, Lesław Szydłowski, Kamila Maria Ludwikowska, Magdalena Okarska-Napierała, Natalia Dudek, Krzysztof Piwoński, Aneta Afelt, Catherine Suski-Grabowski, Miron Bartosz Kursa, Ernest Kuchar, Leszek Szenborn, Teresa Jackowska, Jarosław Peregud-Pogorzelski, Artur Mazur

**Affiliations:** ^1^ Department of Pediatrics and Pediatric Endocrinology, Faculty of Medical Sciences, Medical University of Silesia, Katowice, Poland; ^2^ Department of Pediatrics and Pediatric Endocrinology, John Paul II Upper Silesian Child Health Centre, Katowice, Poland; ^3^ Department of Pediatric Cardiology, Medical University of Silesia, Katowice, Poland; ^4^ Department of Pediatric Infectious Diseases, Wrocław Medical University, Wrocław, Poland; ^5^ Department of Pediatrics with Clinical Assessment Unit, Medical University of Warsaw, Warsaw, Poland; ^6^ Interdisciplinary Centre for Mathematical and Computational Modelling, University of Warsaw, Warsaw, Poland; ^7^ Espace-DEV, IRD - Institut de Recherche pour le Développement, Montpellier, France; ^8^ Department of Pediatrics, Medical Center of Postgraduate Education, Warsaw, Poland; ^9^ Department of Pediatrics and Pediatric Oncology, Pomeranian Medical University, Szczecin, Poland; ^10^ Department of Pediatrics, Pediatric Endocrinology and Diabetes, Medical College University of Rzeszów, Rzeszów, Poland

**Keywords:** obesity, childhood obesity, COVID-19, MIS-C, PIMS

## Abstract

**Background:**

A new disease entity called multisystem inflammatory syndrome in children (MIS-C) is a rare consequence of COVID-19 infection. The pathophysiology and risk factors of MIS-C are still unclear, and the clinical manifestation ranges from milder forms to cases needing intensive care unit treatment. Based on available data, obesity is linked to pro-inflammatory stimulation. Moreover, several studies showed that obesity could play a role in COVID-19 severity and its comorbidities among the adult and children’s populations. This study aimed to investigate the influence of overweightedness/obesity in childhood for the course of MIS-C in Poland.

**Methods:**

This study presented data from the national MultiOrgan Inflammatory Syndromes COVID-19 Related Study (MOIS-CoR) collected between 4 March 2020 and 20 February 2021. Of the 371 patients that met the Polish MIS-C criteria, 306 were included for further analysis.

**Results:**

Children who are obese (OB with body mass index (BMI) ≥95th percentile) and overweight (OV with BMI ≥85th percentile but <95th percentile) (28 and 49 patients, respectively) represented 25.1% (n=77) of all recruited patients. Complete recovery at the time of discharge presented in 93% of normal body weight (NW) participants and 90% of OV children (p>0.05). Among OB children, 76% recovered fully, which differed from the NW group (p=0.01). Calculated odds ratio (OR) of incomplete recovery for OB children was 4.2. Irrespective of body weight, there were no differences (p>0.05) in the length of hospitalization and the duration of symptoms (for OB, 13 and 16.5 days; for OV and NW, 10 and 14 days, respectively), as well as in the frequency of cardiovascular abnormalities, necessity of oxygen therapy (OB, 26.9%; OV, 23.9%; and NW, 20.7%), and intravenous immunoglobulin and glucocorticosteroid (GCS) treatment.

**Conclusion:**

The higher risk of incomplete recovery and observed tendency toward a worsening course of MIS-C in patients with obesity suggest the need for further studies to confirm and understand our findings.

## Introduction

In mid-April 2020, a few cases of uncommon multisystem inflammatory conditions emerged during the COVID-19 pandemic (1). This new disease entity, called, by the Royal College of Pediatrics and Child Health in the United Kingdom, pediatric inflammatory multisystem syndrome temporally associated with severe acute respiratory syndrome coronavirus 2 (PIMS-TS) (2), is described by the World Health Organization and the Centers for Disease Control and Prevention in Europe and the United States as the multisystem inflammatory syndrome in children (MIS-C) (3, 4). MIS-C is a rare consequence of COVID-19 infection and usually affects previously healthy children, rarely with comorbidities, after 4–8 weeks of COVID-19 infection (5).

The pathophysiology and risk factors of the disease are not yet clear, but they mostly included male sex, concomitance of asthma, diabetes mellitus, neurologic disorders, and other chronic disorders like obesity (5). Clinical manifestation ranges from milder forms to cases in need of intensive care unit treatment (6). Patients may present features characteristic of other inflammation entities such as Kawasaki disease (KD) (7, 8), atypical Kawasaki disease (aKD), toxic shock syndrome (TSS) (9, 10), or macrophage activation syndrome (MAS) (11). The abnormalities are usually observed among the cardiovascular, gastrointestinal, dermatological, hematological, neurological, and respiratory systems.

Based on available data, obesity, due to engaging inflammatory pathways and cytokine signaling cascades, may lead to chronic low-grade inflammation (12–14). Although the pathways correlating obesity with SARS-CoV-2 infection are still hypothetical (15, 16), several studies showed that obesity plays a role in COVID-19 severity among adult and children’s populations (5, 17–19).

Here, we analyzed data from the national MultiOrgan Inflammatory Syndromes COVID-19 Related Study (MOIS-CoR) to investigate the influence of overweightedness/obesity on the PIMS-TS/MIS-C course and final outcome.

## Materials and methods

We analyzed the MOIS-CoR database containing data collected between 4 March 2020 and 20 February 2021. The database developments were adopted and described previously (20, 21). The data were collected using a special online form that was created and standardized by a multicenter group of experts from all over the country. The first Polish recommendation for MIS-C was established on April 2020. The criteria for PIMS/MIS-C and other clinical status definitions are presented in **Tables 1**, **2**.

**Table 1:** The Polish inclusion criteria of PIMS/MIS-C (20).

Age 0-18 years old
Need for hospitalization
Time criterion: admission after 4 March 2020
Clinical criteria: Diagnosed with Kawasaki disease (KD) or incomplete (atypical) Kawasaki disease (aKD) or toxic shock syndrome (TSS) or macrophage activation syndrome (MAS) or unspecified inflammatory syndrome
Exclusion of other causes of the disease (infectious or non-infectious)
Laboratory confirmation of COVID-19 (RT-PCR, antigen test, serology) or history of personal exposure with patient with COVID-19 might be positive or negative

**Table 2:** Clinical and biochemical status definitions.

Data:	Definition:
Nutritional status	Expressed in body-mass index (BMI), converted into Z-scores based on the WHO reference standards for children younger than 5 years old and national reference standards for older children (22)
Normal body weight	5th percentile to less than the 85th percentile of BMI for age and sex (WHO reference)
Overweightedness	85th percentile to less than the 95th percentile of BMI for age and sex (WHO reference)
Obesity	Greater than the 95th percentile of BMI for age and sex (WHO reference)
hSDS (height standard deviation score)	Patient’s height – 50th percentile of height for age and sex)/(50th percentile of height for age and sex – 3rd percentile of height for age and sex)*1/2; based on Polish height charts (23)
Heart dysfunction	Left ventricular ejection fraction (LVEF) <55%
Severe heart dysfunction	Left ventricular ejection fraction (LVEF) <35%
Coronary artery Z-score	Based on the Dallaire equation or Boston Children’s Hospital Z-score calculator, in relation to the body surface area (24)
Dilation	Z-score between 2 and 2.5
Aneurysm	Z-score ≥2.5
Elevated BNP/NT-proBNP	>150 ng/ml
Elevated both T and I troponin	Threshold of 50 ng/L
Renal dysfunction	Estimated glomerular filtration rate (eGFR)<90 ml/min/1.73 m2, calculated using the revised Schwartz formula (25)
Hypoproteinemia	Total protein count <6.0 g/dL
Hypoalbuminemia	Serum albumin <3.5 g/dL
Elevated alanine transaminase	≥40 U/L
Elevated aspartate transaminase	≥45 U/L
Elevated gamma-glutamyltransferase (GGT)	≥22 U/L
Elevated lactate dehydrogenase (LDH)	≥380 U/L
Elevated C-reactive protein (CRP)	≥10 mg/L
Elevated procalcitonin	≥0.5 ng/ml
Elevated triglyceride (TG)	≥125 U/L
Elevated interleukin 6 (IL-6)	>7 pg/ml
Hyponatremia	Serum sodium <135 mmol/L
Thrombocytopenia	Platelets <150 ×109/L,
Lymphopenia	Lymphocyte count <1.5 ×109/L
Leukocytosis	Leukocyte count >12 ×109/L
Neutrophilia	Neutrophil count >7 ×109/L
Neutrophil-lymphocyte ratio/NLR	Age-dependent reference values
AVPU scale	Level of consciousness defined as: A-Alert (patient is awake); V-Verbal (patient responds to a verbal stimulus); P-Pain (patient responds to a pain stimulus); and U-Unresponsive (patient is unresponsive to stimulus)
Full recovery at the time of discharge	Subjective opinion of the pediatric specialist or/and lack of cardiac complications

### Statistical methods

All statistical analyses were performed using Statistica 13 PL software (StatSoft Inc., Tulsa, OK, USA). A p-value of <0.05 was considered significant. The Shapiro-Wilk test was utilized to verify the normality of distribution. To compare qualitative variables, the Chi^2^ test was utilized. Odds ratio was used for logistic regression. The comparisons between two parametric values were made by using the Student’s t-test or the Mann-Whitney U test for non-parametric distributions.

## Results

### Clinical phenotype

**Figure 1:**
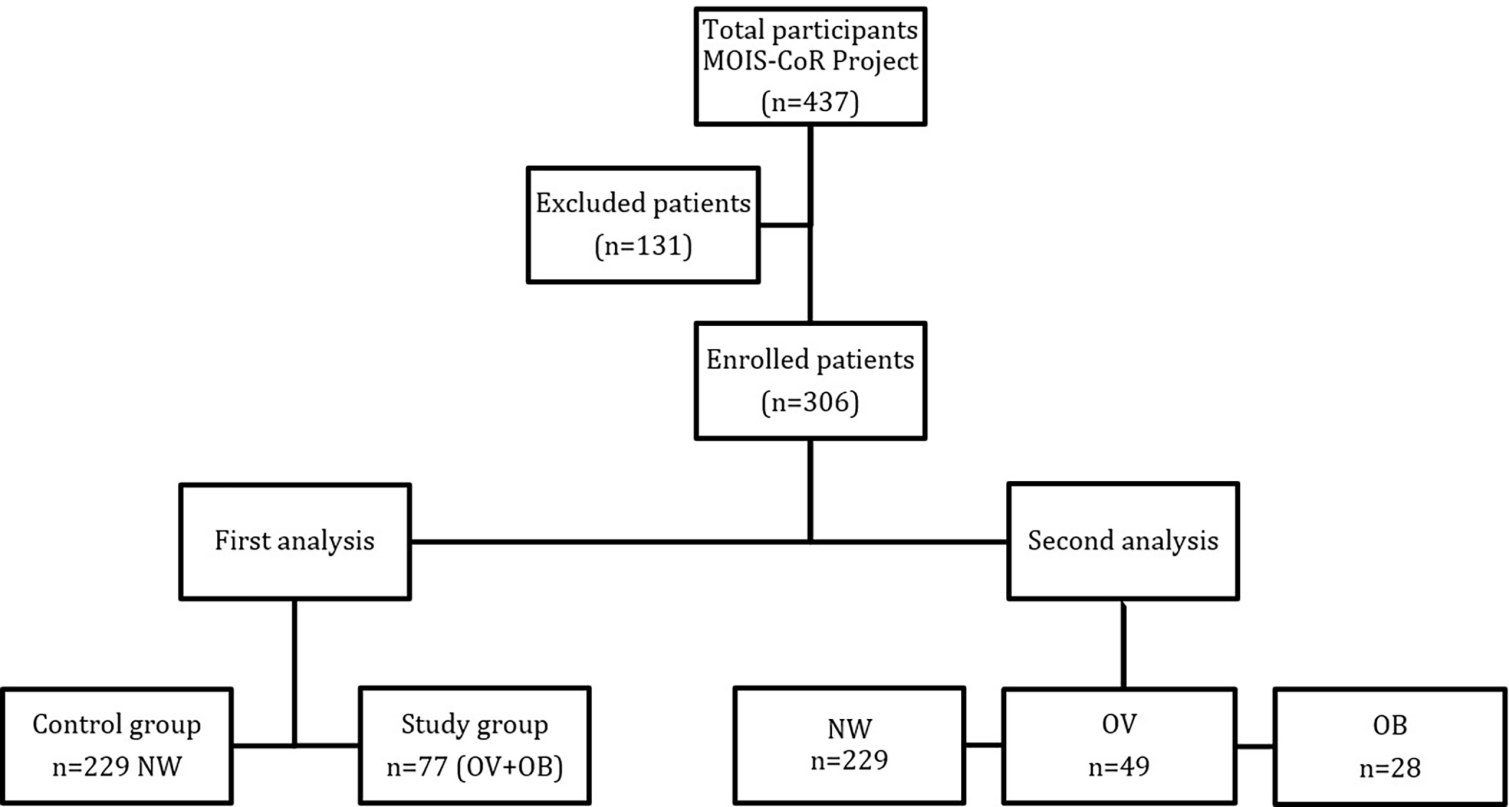
Flowchart of patients included in the MOIS-CoR study.

The MOIS-Cor Project included 437 patients. Our population, except for two Asian patients, was homogeneous, and comprised Caucasian children. Twelve patients did not fulfill the Polish PIMS criteria. Additionally, 54 cases were excluded due to lack of data. The Polish PIMS criteria were fulfilled by 371 patients. Moreover, to compare children with obesity/overweightedness to children with normal body weight, 22 underweight children were excluded. Of the 349 remaining patients, 43 were also excluded due to missing values in age, sex, height or weight. In further analyses, 306 patients were included. Two separate analyses were conducted: the first one included a study group (Obese/OB + Overweight/OV; n=77) vs. a control group (Normal Weight/NW; n=229), and the second encompassed all three groups: NW vs. OB vs. OV patients. The flowchart is presented in **Figure 1**.

There were 229 NW, 28 OB, and 49 OV patients. They were 8.18 (95% CI: 7.6; 8.77), 8.55 (95% CI: 6.73; 10.38), and 8.47 (95% CI: 7.14; 9.81) years old, respectively. OB and OV children represented 25.1% (n=77) of all recruited patients. These groups did not differ in age, sex, and height as well as in the occurrence of other comorbidities (p>0.05) (**Table 3**).

**Table 3:** Anthropometrical data of study subjects.

	Normal (n=229)	Overweight (n=49)	Obese (n=28)	P-value
Age [y]	8.2 (7.6; 8.8)	8.6 (6.7; 10.4)	8.5 (7.1; 9.8)	0.89
Height [cm]	129.4 (125.7; 133.2)	132.9 (123.4; 142.4)	134.5 (121.8; 141.1)	0.58
hSDS	0.4 (0.2; 0.6)	0.5 (0.1; 0.9)	0.9 (0.2; 0.6)	0.6
Weight [kg]	30.4 (28.3; 32.6)	41.1 (34.7; 47.5)	52.6 (40.5; 64.8)	<0.001
BMI Z-Score*	-0.3 (-0.8; 0.4)	1.3 (1.2; 1.5)	2.2 (1.9; 2.4)	<0.001
	**Control (n=229)**	**Study (n=77)**		
Age [y]	8.2 (7.6; 8.8)	8.5 (7.5; 9.6)		0.59
Height [cm]	129.4 (125.7; 133.2)	133.4 (126.0; 140.9)		0.31
hSDS	0.4 (0.2; 0.6)	0.7 (0.3; 1.0)		0.46
Weight [kg]	30.4 (28.3; 32.6)	45.3 (39.3; 51.3)		<0.001
BMI Z-Score*	-0.3 (-0.8; 0.4)	1.5 (1.3; 2.0)		<0.001

Data presented as mean (95% CI); *presented as median (IQR); hSDS, height standard deviation score; BMI Z-score, body mass index Z-score.

There were 5.6% (n=17/306) of patients categorized below “A” in the alert-verbal-pain-unresponsive (AVPU) scale at admission. In regard to body weight, n=2/49 (4.1%) were OV, n=15/214 (6.5%) were NW, and none was OB. During hospitalization, n=2/23 (7.1%) of OB children, n=6/44 (12.2%) of OV children, and n=37/207 (16.2%) of NW patients were described as below “A” in the AVPU scale.

### Biochemical phenotype

Comparisons of laboratory tests results are presented in **Tables 4**, **5**. Apart from interleukin 6 (IL-6) and total protein at admission, there was no difference between the study group (OV and OB) and the control group in inflammatory assays such as C-reactive protein (CRP), procalcitonin, leukocytosis, neutrophilia, lymphopenia, neutrophil–lymphocyte ratio (NLR), and albumins.

**Table 4:** Comparison of laboratory results between the control and study groups.

	Control group (n=226)	Study group (n=77)	P-value
CRP at adm [mg/L]	142.6 ± 94.9	160.3 ± 104.6	0.22
CRP at max [mg/L]	171.1 ± 110.1	181.6 ± 111.7	0.41
PCT at adm [mg/L]	7.8 ± 14.3	7.2 ± 19.5	0.26
PCT at max [mg/L]	12.0 ± 22.8	11.3 ± 24.1	0.07
D-dimers at adm [mg/L]	4.3 ± 8.2	4.1 ± 5.7	0.53
NT-proBNP at adm [pg/ml]*	2,868.5 (681.4; 6,621.5)	1,010.0 (173.0; 5,683.0)	0.17
NT-proBNP at max [pg/ml]*	4,809.5 (2,337.0; 11,313.0)	5,707.0 (993.0; 15,448.5)	0.83
Ferritin at adm [µg/L]	979.8 ± 4,068.5	931.4 ± 3,619.9	0.69
Ferritin at max [µg/L]*	401.0 (211.7; 661.0)	403.0 (198.0; 779.0)	0.88
WBC at adm [103/µl]	11.3 ± 6.9	11.8 ± 6.0	0.28
WBC at max [103/µl]	16.2 ± 7.6	17.8 ± 7.9	0.09
Neutrophils at adm [103/µl]	9.3 ± 12.0	9.3 ± 5.2	0.19
Lymphocytes at adm [103/µl]	1.7 ± 1.8	1.8 ± 1.9	0.87
Lymphocytes at min [103/µl]	1.6 ± 1.5	1.6 ± 1.5	0.96
NLR at adm	8.7 ± 8.6	9.1 ± 7.6	0.58
Albumins at adm [g/dl]	3.3 ± 0.6	3.5 ± 7.6	0.08
Albumins at min [g/dl]	3.0 ± 0.6	2.9 ± 0.6	0.73
INR	1.3 ± 0.2	1.3 ± 0.2	0.08
APTT [s]	35.4 ± 6.7	35.6 ± 8.4	0.60
AspAT [U/L]	62.0 ± 194.6	74.3 ± 90.6	0.16
AlAT [U/L]	49.2 ± 124.8	70.1 ± 89.5	0.002
Plasma bilirubin [mg/dl]	1.19 ± 3.4	1.2 ± 1.5	0.09
Sodium [mmol/L]	134.1 ± 4.2	135.6 ± 3.8	0.03
Glucose [mg/dl]	105.5 ± 25.6	101.3 ± 25.3	0.20
LDH [U/L]	344.8 ± 458.6	399.4 ± 491.1	0.08
Triglicerydes [mg/dl]	172.5 ± 84.4	169.3 ± 79.3	0.80
Amylase [U/L]	35.5 ± 30.7	35.2 ± 22.9	0.73
Serum creatinine [mg/dl]	0.61 ± 0.61	0.7 ± 0.8	0.09
GGTP [U/L]	40.7 ± 45.5	104.2 ± 116.0	<0.001
IL-6 [pg/ml]	324.0 ± 645.5	114.0 ± 219.2	0.01
Total protein at adm [g/dl]	5.8 ± 1.1	6.3 ± 1.0	0.004
Total protein at max [g/dl]	5.9 ± 1.2	5.9 ± 1.1	0.23
BNP at adm [pg/ml]*	319.0 (134.9; 1012.0)	184.4 (108.0; 730.0)	0.30
BNP at max [pg/ml]*	2,299.4 (443.5; 9502.0)	589.1 (156.1; 3,707.7)	0.01

*Data presented as median (IQR, interquartile range), other data presented as mean ± standard deviation; at adm, measured at admission; at max, maximum level during hospitalization; at min, minimum level during hospitalization; CRP, C-reactive protein; PCT, procalcitonin; NT pro-BNP, N terminal prohormone of brain natriuretic peptide at admission; WBC white blood count; NLR, neutrophil–lymphocyte ratio; INR, international normalized ratio; APTT, activated partial thromboplastin time; AspAT, aspartate transaminase; AlAT, alanine transaminase; LDH, lactate dehydrogenase; IL-6, interleukin 6; BNP, brain natriuretic peptide.

**Table 5:** Comparison of laboratory results between the NW, OV, and OB groups at admission.

	NW (n=226)	OV (n=49)	OB (n=28)	P-value
CRP at adm [mg/L]	142.6 ± 94.9	143.5 ± 92.8	190.8 ± 119.1	0.12
CRP at max [mg/L]	171.1 ± 110.1	171.6 ± 100.1	197.9 ± 128.1	0.60
PCT at adm [mg/L]	7.8 ± 14.3	6.0 ± 20.9	9.3 ± 17.1	0.28
PCT at max [mg/L]	12.0 ± 22.8	8.6 ± 22.2	15.9 ± 27.0	0.08
D-dimers at adm [mg/L]	4.3 ± 8.2	3.8 ± 6.0	4.6 ± 5.3	0.67
NT-proBNP at adm [pg/ml]*	2868.5 (681.4; 6621.5)	833.2 (313.5; 2,974.0)	3061.0 (167.0;19064.0)	0.13
NT-proBNP at max [pg/ml]*	4809.5 (2,337.0; 11313.0)	5,406.0 (993.0; 9,734.0)	9372.5 (2025.5; 18416.5)	0.67
Ferritin at adm [µg/L]	979.8 ± 4068.5	1198.3 ± 4501.3	448.4 ± 371.6	0.77
Ferritin at max [µg/L]*	401.0 (211.7; 661.0)	409.5 (197.5; 1113.0)	358.0 (202.9; 589.0)	0.74
WBC at adm [103/µl]	11.3 ± 6.9	11.6 ± 6.2	12.1 ± 5.8	0.46
WBC at max [103/µl]	16.2 ± 7.6	17.2 ± 6.9	18.9 ± 9.4	0.21
Neutrophils at adm [103/µl]	9.3 ± 12.0	9.3 ± 5.4	9.3 ± 4.8	0.39
Lymphocytes at adm [103/µl]	1.7 ± 1.8	1.7 ± 1.8	1.9 ± 2.3	0.97
Lymphocytes at min [103/µl]	1.6 ± 1.5	1.6 ± 1.6	1.5 ± 1.3	0.96
NLR at adm	8.7 ± 8.6	9.3 ± 8.1	8.6 ± 6.9	0.83
Albumins at adm [g/dl]	3.3 ± 0.6	3.46 ± 0.6	3.46 ± 0.6	0.21
Albumins at min [g/dl]	3.0 ± 0.6	2.9 ± 0.6	3.0 ± 0.7	0.78
INR	1.3 ± 0.2	1.3 ± 0.2	1.3 ± 0.3	0.19
APTT [s]	35.4 ± 6.7	35.0 ± 6.6	36.6 ± 11.1	0.87
AspAT [U/L]	62.0 ± 194.6	68.5 ± 87.7	84.6 ± 96.6	0.31
AlAT [U/L]	49.2 ± 124.8	56.3 ± 61.5	94.1 ± 121.6	0.003
Plasma bilirubin [mg/dl]	1.19 ± 3.4	1.2 ± 1.4	1.3 ± 1.9	0.13
Sodium [mmol/L]	134.1 ± 4.2	135.6 ± 4.0	135.6 ± 3.4	0.08
Glucose [mg/dl]	105.5 ± 25.6	102.0 ± 25.6	135.6 ± 25.1	0.43
LDH [U/L]	344.8 ± 458.6	427.8 ± 615.7	353.7 ± 151.7	0.14
Triglycerides [mg/dl]	172.5 ± 84.4	166.3 ± 76.9	176.4 ± 87.4	0.87
Amylase [U/L]	35.5 ± 30.7	32.1 ± 22.4	40.1 ± 23.7	0.41
Serum creatinine [mg/dl]	0.61 ± 0.61	0.66 ± 0.7	0.69 ± 0.70	0.22
GGTP [U/L]	40.7 ± 45.5	106.2 ± 125.6	99.9 ± 96.3	<0.001
IL-6 [pg/ml]	324.0 ± 645.5	80.3 ± 79.6	195.1 ± 403.9	0.04
Total protein [g/dl]	5.8 ± 1.1	6.1 ± 0.9	6.6 ± 1.1	0.01
Total protein at max [g/dl]	5.9 ± 1.2	5.9 ± 1.0	6.0 ± 1.1	0.48
BNP at adm [pg/ml]*	319.0 (134.9; 1012.0)	230.1 (134.9; 850.0)	129.7 (287.2; 318.6)	0.28
BNP at max [pg/ml]*	2299.4 (443.5; 9502.0)	589.1 (179.5; 2918.6)	606.8 (58.2; 21524.5)	0.04

*Data presented as median (IQR, interquartile range), other data presented as mean ± standard deviation; at adm, measured at admission; at max, maximum level during hospitalization; at min, minimum level during hospitalization; CRP, C-reactive protein; PCT, procalcitonin; NT pro-BNP, N terminal prohormone of brain natriuretic peptide at admission; WBC, white blood count; NLR, neutrophil–lymphocyte ratio; INR, international normalized ratio; APTT, activated partial thromboplastin time; AspAT, aspartate transaminase; AlAT, alanine transaminase; LDH, lactate dehydrogenase; IL-6, interleukin 6; BNP, brain natriuretic peptide.

There were significant differences between transaminases (AlAT, GGT) in the control/NW and the OB/OV groups, but no difference in the case of AspAT, LDH, and TG (**Tables 4**, **5**).

Brain natriuretic peptide (BNP) level at admission did not differ between the control/NW and study/OB and OV; however, the BNP level at its maximum was higher in the control group (**Tables 4**, **5**).

### Cardiac phenotype/status

Those who had some echocardiographic abnormalities (decreased left ventricular ejection fraction (LVEF), coronary artery abnormalities (CAA) or pericardial effusion) were 40.6% (n=26/64) of the study/OB and OV patients and 29.4% (n=58/197) of the control/NW patients; however, there were no differences in particular cardiovascular abnormalities between groups (p>0.05). Those who developed cardiac disorders (p=>0.05) were 47.6% (n=10/21) of OB, 37.2% (n=16/43) of OV, and 29.4% (n=58/197) of NW patients. Among the study/OB and OV patients, 17.2% (n=11/64) were reported with decreased left ventricular ejection fraction during hospitalization, and 10.0% (n=5/50) maintained this condition. Pericardial effusion occurred in 9.4% (n=6/64) of the study/OB and OV patients, with two cases of arrhythmia and one case of myocarditis. There were 12.8% (n=5) of the study/OB and OV patients who developed vessel malformations (two cases of dilatation, one small aneurysm, one medium aneurysm, and one large aneurysm; see **Table 6**).

**Table 6:** Clinical findings of control and study groups.

	Control group	Study group	p-value
Complete recovery at time of discharge	93.0% (n=185)	85.1% (n=57)	0.051
Length of hospitalization [days]	10; (8;13)	11 (8;14)	0.77
Duration of symptoms [days]	14; (12; 17.5)	14.5 (10;17)	0.57
Chest X-ray or CT abnormalities	19.0% (n=40)	20.3% (n=14)	0.80
Echocardiographical abnormalities	29.4% (n=58)	40.6% (n=26)	0.10
Decreased LV EF	17.8% (n=35)	17.2% (n=11)	0.72
Preserved decreased LV EF	9.9% (n=16)	10% (n=5)	0.97
Pericardial effusion	9.1% (n=18)	9.4% (n=6)	0.95
CAA	4.59% (n=9)	10.2% (n=4)	0.23
Respiratory failure	20.7% (n=46)	25.0% (n=18)	0.44
Hospitalization in PICU	8.8% (n=20)	6.6% (n=5)	0.54
Length of PICU hospitalization*	6.5 (2; 39)	6 (3; 7)	0.27
Mechanical ventilation	4.1% (n=9)	2.7% (n=2)	0.61
IVIG administered	88.0% (n=198)	92.0%(n=69)	0.34
Glucocorticoids administered	65.1% (n=136)	72.6% (n=53)	0.24
Preventive dosage of heparin	24.7% (n=48)	29.5% (n=18)	0.70
Therapeutic dosage of heparin	10.3% (n=20)	11.5% (n=7)	0.70
ASA	85.3% (n=193)	92.0% (n=69)	0.35

Data presented as percentage (quantity) or median (IQR); LVEF, left ventricular ejection fraction; CAA, coronary artery abnormalities; PICU, pediatric intensive care unit; IVIG, intravenous immunoglobulin; ASA, acetylsalicylic Acid.

A total of 34.0% (n=104/306) of patients developed hypotension during hospitalization; 10 (35.7% n=10/28) of the OB, 17 (34.7% n=17/49) of the OV, and 77 (33.6% n=77/229) of the NW groups (p>0.05).

### Additional exam(s)

A total of 17.6% (n=54/306) of patients had detected X-ray or CT abnormalities at any time of hospitalization: In the OB group, it was 28.6% (n=8/28); in OV, 12.2% (n=6/38); and in NW, 17.5% (n=40/229). Only 75.9% (n=41/54) of all patients with chest imaging abnormalities recovered fully at the time of discharge. Two out of eight (25%) OB, one out of six (16.7%) OV, and three out of 40 (7.5%) NW children did not achieve full recovery.

### Treatment

Oxygen supplementation was necessary in 25.0% (n=18) of cases in the study/OV and OB patients and in 20.7% (n=46) in the control/NW group (p>0.05). Oxygen therapy was required by 26.9% (n=7/26) of OB, 23.9% (n=11/46) of OV, and 20.7% (n=46/222) of NW patients (p>0.05). Among all participants, 5.7% (n=25) needed hospitalization at the pediatric intensive care unit (PICU); three of them were obese, and two were overweight. A total of 6.79% (n=11) of patients required mechanical ventilation, and two of them were from the study group. Of the mechanically ventilated patients, 72.7% (n=8/11) achieved full recovery. The median duration of hospitalization at the PICU was 6 days (interquartile range, IQR: 4-7), while the longest hospitalization at the PICU was 39 days. Among all patients who needed oxygen supplementation, 5.3% (n=3/53) did not achieve full recovery. All patients with obesity who were treated with oxygen reached full recovery at the time of discharge. Length of hospitalization was 13 (IQR: 10-16) days, and the symptoms lasted 17 (IQR: 15-26) days. A total of 53.1% (n=34/64) of the patients had diagnosed echocardiographic abnormalities. Intravenous immunoglobulin (IVIG) was administered to 87.3% of patients (n=267): 92.0% (n=69) of the study/OB and OV and 88.0% (n=198) of the control/NW patients (p>0.05). Glucocorticosteroids (GCSs) were administered in 72.6% (n=53) of the study/OB and OV patients and 65.1% (n=136) of the control/NW groups (p>0.05). A preventive dose of heparin s.c. was used in 29.5% (n=18) of the children in the study/OB and OV group and 24.7% (n=48) in the control/NW group (p>0.05). There were also no differences in the usage of a therapeutic dosage: 11.5% (n=7) and 10.3% (n=20) in the study and control groups, respectively. Acetylsalicylic acid (ASA) was administered to 85.6% (n=262) of the patients: 92.0% (n=69) of study patients and 85.8% (n=193) of normal body weight patients (p>0.05). There were no differences in the length of hospitalization and the duration of symptoms between the study and control groups (p>0.05). In the study group, the median length of hospitalization was 11 days (IQR: 8-14) and the symptoms lasted 14.5 days (IQR: 10-17). OB patients needed 13 (IQR: 9-16) days of hospitalization. OV patients and those with normal weight (NW) were equally hospitalized for 10 (IQR: 8-13) days. All three groups did not differ between each other (p>0.05). Symptoms lasted 16.5 (IQR: 12-19) days for the OB group, 14 (IQR: 11-17) days for the OV group, and 14 (IQR: 12-17) days for the NW group. There was no difference between groups (p>0.05). Data were not available on whether patients who did not recover after discharge were discharged to home or referred to another hospital unit.

**Figure 2:**
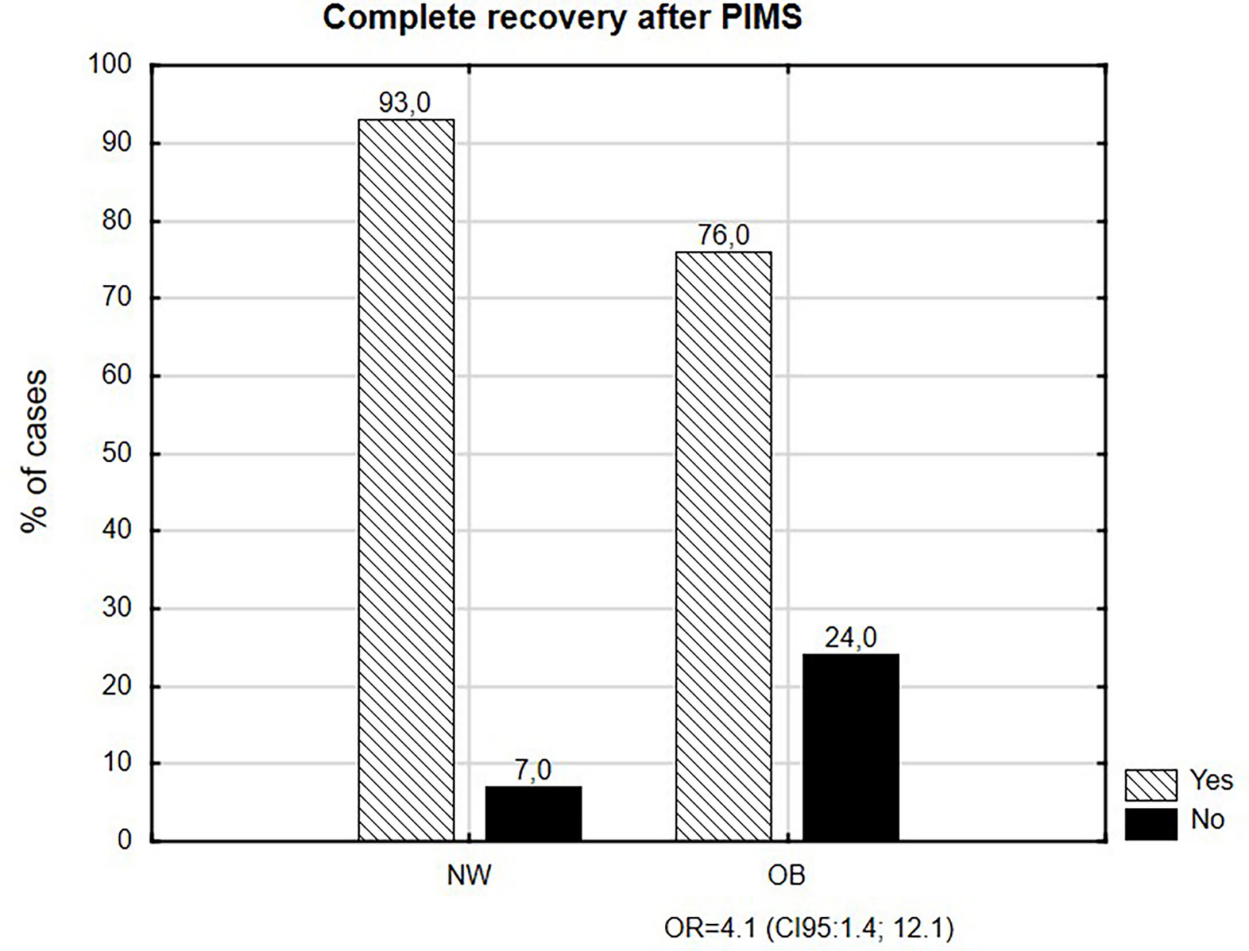
Complete recovery after PIMS.

Body weight did not differentiate the study group/OB and OV from the control group in regard to complete recovery at the time of discharge: 85.1% and 93.0%, respectively (p=0.06). However, only 76.0% (n=19/25) of OB children recovered fully and differed from the NW group (p=0.01; see **Figure 2**). Calculated odds ratio of incomplete recovery for OB children was 4.2 (95% CI: 1.4; 12.1) (**Table 4**). Among the OV children, 90% (n=38/42) recovered fully and this result did not differ compared to NW and OB children (p>0.05).

## Discussion

Pediatric inflammatory multisystem syndrome temporally associated with SARS-CoV2, known as multisystem inflammatory syndrome in children (PIMS-TS/MIS-C), is a rare disorder, sometimes followed by extremely severe conditions and usually occurring in previously healthy children about 4–8 weeks after the onset of COVID-19 infection. According to available publications, obesity and its comorbidities have influence on the severity of COVID-19 infection among adult and pediatric populations (5, 17, 18, 26, 27) and might also be a risk factor for death, next to immunosupression, diabetes mellitus, chronic lung disease, and cardiac and neurological disorders (5). Possible pathways explaining this connection, such as overexpression of angiotensin 2 and increased production of inflammatory cytokines such as TNF-α, IL-1, and IL-6 by adipose tissue, are still not confirmed (27, 28).

In our article, we have analyzed the impact of obesity and overweightedness among Polish children on the severity and final outcome of PIMS-TS/MIS-C.

Studies from other countries mainly focused on describing the frequency of overweightedness and obesity among MIS-C/PIMS-TS populations (29–32). A systematic review in the US revealed a 50.8% rate of overweight or obese patients (vs. 25.1% in our study) among children with comorbidities, and indicated a need for further research among that group (30). Another study described the percentage of obesity to be higher, compared to our results (up to 26% vs. 9.1%) (29, 33). The lower prevalence might be a consequence of the demographic characteristic of Polish children. Depending on age and gender, the obesity rate in Polish adolescents and children is 13–14% (34). Secondly, the databases used were relatively wide-ranging (n=662, n=1080 patients included vs. n=306 in our research) and outlined the ethnic diversity among patients, such as a high prevalence of Hispanic and non-Hispanic black patients in contrast to the representation of Caucasian children in a homogeneous Polish population (20, 21, 29, 30, 33).

Moving to the laboratory results in our study group (OB/OV), we noticed the significant increase of transaminases (AlAT, GGT), which might be explained by liver diseases/non-alcoholic fatty liver disease (NAFLD) spectrum in children with obesity. On the other hand, there was no essential increase in inflammatory assays to support a hypothesis of chronic hepatitis.

Interestingly, the level of IL-6 differed significantly between groups (324 pg/ml for NWs vs. 80.3 pg/ml in OV and 195 pg/ml in OB). The level of interleukin 6, according to available research, increases both in MIS-C and obesity (4, 12, 27, 35). Because of that, we assumed multiplied elevation of IL-6, but our result was opposite to the hypothetical model. In this context, the mechanism of IL-6 elevation as well as higher maximal values of BNP in the control group with normal weight remained unknown and required further studies.

Moving forward in reference to a US surveillance study, obesity (up to 39% vs. 9.1% in obesity and 6.0% vs. 16.0% in overweightedness) was linked to decreased cardiac function (31), contrary to our findings that did not confirm a difference between the study and control groups in cases of cardiovascular abnormalities, including decreased left ventricular ejection fraction. This might be explained by a tendency toward a milder course of the disease in our population (20, 21); however, it demands further investigation.

The administration of intravenous immunoglobulins, steroids, heparin, and acetylsalicylic acid did not differ between groups and was similar to other studies (29, 30, 33) as well as the length of hospitalization. The tendency to a more frequent use of oxygen therapy was noticed in the groups with overweightedness and obesity.

Multicenter MOIS-CoR database analysis confirmed that obesity was less commonly predisposed to a complete recovery at the time of discharge after MIS-C diagnosis, compared to normal-weight patients (76% vs. 93%). Our findings showed that obesity increased the risk of incomplete recovery at the time of discharge by four times. To our best knowledge, this is the first report of an increased risk of incomplete recovery connected with a body mass index above the 95^th^ percentile. This might suggest a tendency to worsen the course of MIS-C. To understand our findings, we need to continue and expand surveillance.

### Study limitations

Although the MOIS-CoR project was created by a multicenter group of pediatric specialists, and the acquisition of the database was standardized, not all the data were collected from all the patients. Moreover, some patients were transferred between centers from low to high referentiality, and because of that, some parameters at admission might not reflect the values at the beginning of the disease. Lastly, the analyzed data, ‘recovery at the time of discharge’, depend on the subjective opinion of the pediatric specialist and/or lack of cardiac complications.

## Data availability statement

The original contributions presented in the study are included in the article/supplementary material. Further inquiries can be directed to the corresponding author.

## Ethics statement

This study was reviewed and approved by the Bioethics Committee at the Wrocław Medical University Poland (CWN UMW BW: 313/2020). All research was performed in accordance with relevant guidelines and regulations. The Bioethics Committee at Wroclaw Medical University granted waiver of informed consent, as only de-identified data were transmitted and analyzed.

## Author contributions

Conceptualization: AG, EB-S, IC, and AM; methodology: AG, ML, EB-S, and IC; validation: AG and EB-S; formal analysis: AG and ML; investigation and resources: EB-S, MF-P, LSzy, KL, MO-N, ND, KP, AAn, AAf, CS-G, MK, EK, LSze, TJ, JP, and AM; data curation: AG; writing: original draft preparation, AG, EB-S, IC, and ML; visualization, ML and IC; supervision, AG and EB-S; project administration, AG. All authors approved the submitted version.

## Funding

The costs of publication were sponsored by the Polish Paediatric Society.

## Conflict of interest

The authors declare that the research was conducted in the absence of any commercial or financial relationships that could be construed as a potential conflict of interest.

## Publisher’s note

All claims expressed in this article are solely those of the authors and do not necessarily represent those of their affiliated organizations, or those of the publisher, the editors and the reviewers. Any product that may be evaluated in this article, or claim that may be made by its manufacturer, is not guaranteed or endorsed by the publisher.
